# These feet were made for walking

**DOI:** 10.7554/eLife.22886

**Published:** 2016-12-14

**Authors:** William L Jungers

**Affiliations:** 1 Department of Anatomical Sciences, Stony Brook University School of Medicine, New York, United Stateswilliamjungers@gmail.com; 2 Association Vahatra, Antananarivo, Madagascar

**Keywords:** Australopithecus afarensis, Hominini, Laetoli, footprints, Pliocene, body size estimates, Other

## Abstract

New fossil footprints excavated at the famous Laetoli site in Tanzania suggest that our bipedal ancestors had a wide range of body sizes.

**Related research article** Masao FT, Ichumbaki EB, Cherin M, Barili A, Boschian G, Iurino DA, Menconero S, Moggi-Cecchi J, Manzi G. 2016. New footprints from Laetoli (Tanzania) provide evidence for marked body size variation in early hominins. *eLife* **5**:e19568. doi: 10.7554/eLife.19568

Walking on two hind limbs, or bipedalism, is one of the defining characteristics of the evolutionary lineage that gave rise to modern humans. Though fragments of fossilized bones suggest that this adaptation might date as far back as 7 million years ago (Zollikofer et al., 2005), this interpretation remains controversial. The earliest unequivocal evidence of bipedalism comes not from bones, but from footprints made some 3.66 million years ago and preserved at a site in Laetoli, Tanzania (Leakey and Hay, 1979). However, it is widely agreed that bipedalism most likely evolved in an ancestor whose brain was no bigger than that of a chimpanzee, and who had not yet started to make and use tools.

The footprints preserved at Laetoli are what are known as "trace fossils", because they are traces of behavior rather than the petrified remains of actual body parts. The footprints were formed when three of our distant hominin relatives – most likely members of the species *Australopithecus afarensis* (White and Suwa, 1987) – walked in the same direction across wet volcanic ash. Millions of years later, in 1976, their preserved footprints were discovered by British paleoanthropologist Mary Leakey and co-workers, and the prints were fully excavated by 1978. Since then, the scientific and public interest in the Laetoli footprints has been extraordinary. They are mentioned in hundreds, if not thousands, of scientific works, and a Google search for "Laetoli footprints" returns 52,600 hits.

Now, in eLife, Marco Cherin of the University of Perugia and Sapienza University of Rome and colleagues – who are based at institutions in Tanzania and Italy – report the discovery of a second set of preserved footprints from Laetoli (Masao et al., 2016). These new trace fossils are the same age as the first ones, and were found at a site called "Site S", which is 150 meters south of "Site G", where the original discovery was made.

Cherin and colleagues – who include Fidelis Masao of the University of Dar es Salaam in Tanzania as first author – present captivating graphics and photographs of the new footprints and describe their setting (Figure 1). The tracks at both Site G and Site S are well preserved in the same hardened volcanic ash known as the "Footprint Tuff" on the southern edge of the Serengeti Plains. It appears that the environment when the footprints were made was not unlike what is seen in this region today – a mix of bushland, woodland and grassland with a nearby forest along the river. Footprints from a rhinoceros, a giraffe, some prehistoric horses and guinea fowl were found at the site. However, the new hominin footprints are most definitely the star attractions. They were left by two individuals – referred to as S1 and S2 – who again were most likely *A. afarensis.*

**Figure 1.**
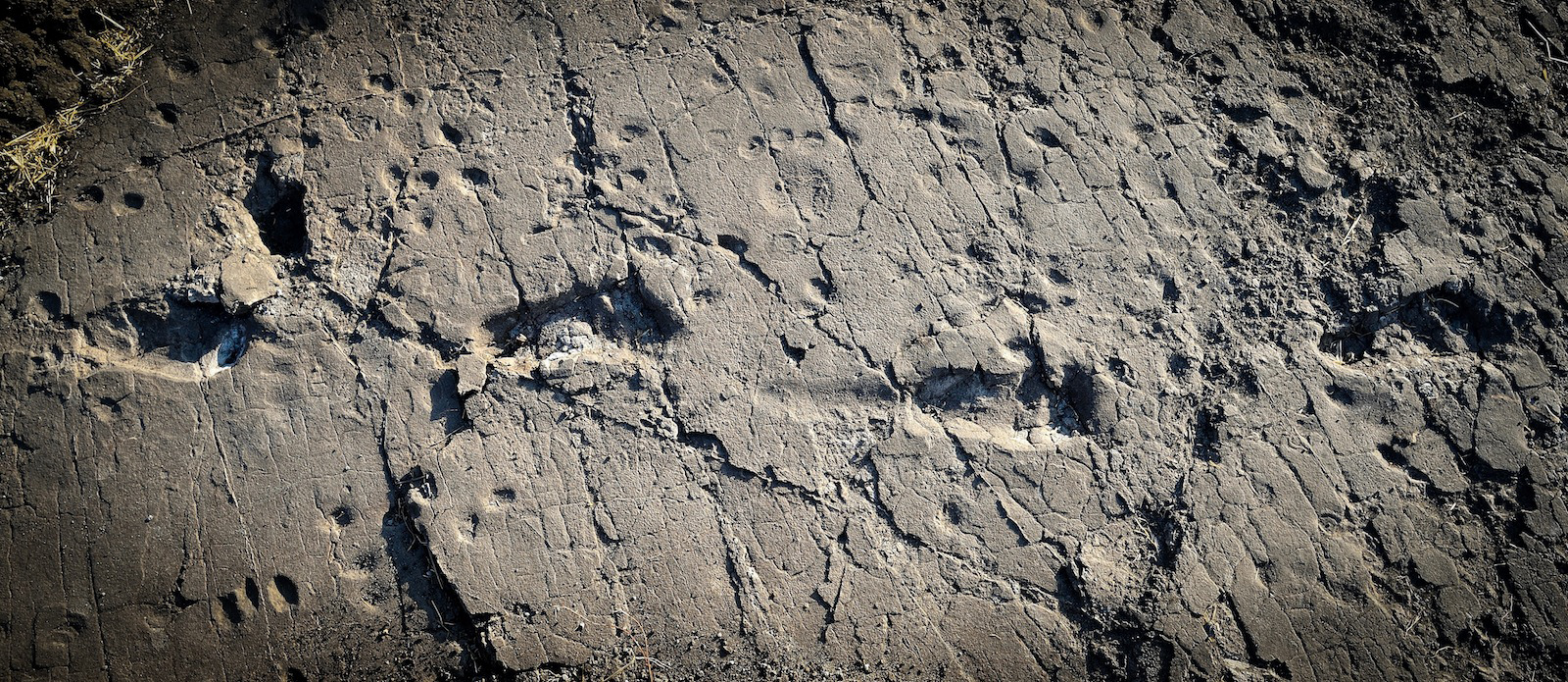
Footprints from an early hominin have been unearthed at a new site in Laetoli, Tanzania. These four footprints shown are thought to have been left by an *Australopithecus afarensis* around 3.66 million years ago, in the Pliocene Epoch. Masao et al. estimate that the individual who left these footprints in the wet volcanic ash was likely taller and heavier than those that left the prints previously discovered at Laetoli.

Like the trace fossils at Site G, the newly found footprints follow a path that heads north-northwest. However, only the multiple footprints from S1 are especially informative; S2 is known from just a single print that is abnormal due to apparent slipping. When photographs of the S1 prints were carefully compared to casts of footprints from Site G, the two sets appeared to be similar in many respects. For example, the heel impressions are deep and oval, and the big toes are in line with the other toes.

So far, Masao et al. have only described the features of the prints and analyzed how weight was transferred through the foot in a qualitative manner that is consistent with the earlier interpretation of the footprints at Site G (Robbins, 1987). Although the big toe appears to be the longest digit, it was not always where most force was applied when the foot pushed off from the ground (as tends to be the case for modern humans). Instead, the deepest impression in some footprints (indicating the most force) occurred more to the side of the foot. Individual toes cannot be distinguished easily from the prints, but a clear ridge was formed across the footprint when the toes gripped the wet volcanic ash and pushed it backward.

At this stage of their analysis, Masao et al. have chosen not to weigh in on the debate over how similar the footprints are to those of modern humans. Previously, some have taken the footprints at Site G as early evidence that *A. afarensis* walked in a remarkably human-like manner (e.g., Raichlen et al., 2010; Crompton et al., 2012). Others have contested this conclusion (e.g., Meldrum et al., 2011). In fact, a recent analysis strongly suggested that the Laetoli footprints were significantly different from those of a modern human walking barefoot, and actually in some ways more similar to chimpanzee footprints (Hatala et al., 2016).

Masao et al. also report several important measurements including the length and width of the prints, the angle of gait, and the step and stride lengths. Based in part on these measurements, they predicted the weight and height (or body mass and stature) of the individuals who left the footprints at the two sites. The prints at Site S were most likely left by individuals who were taller and heavier than any of the three that left prints at Site G. Of the five individuals, the lightest one left footprints at Site G and likely weighed 28.5 kg, while the heaviest walked at Site S and is estimated to have weighed as much as 48.1 kg.

These estimates for body mass fit comfortably within the wide interval calculated for this species based upon fossils of its limb bones (Grabowski et al., 2015). The predicted maximum height for the S1 individual is a different matter and is surprisingly tall at roughly 165 cm. Masao et al. interpret their new data as indicating that different *A. afarensis* individuals might have had very different body sizes. Variation of this magnitude could imply big differences between males and females – a phenomenon referred to as "sexual dimorphism". However, this would only be the case if we assume that all the footprints are from adults, and not if younger individuals made some of the smaller ones.

Nevertheless, and as Masao et al. acknowledge, the height estimates depend on a previously reported relationship between foot length and stature (Dingwall et al., 2013). It is important to note that foot length has only been roughly estimated for this ancient species, and that the height estimates would change if a different foot length-to-stature ratio was used. For example, *Homo floresiensis –* a species of ancient hominid from Indonesia (which have been commonly referred to as "hobbits"; Jungers et al., 2009) – had a different ratio, and if this was used instead, the estimates for the height of the tallest individual at Laetoli (S1) would shrink down to 132–148 cm. Furthermore, the shortest (called G1) would become even shorter at approximately 100 cm: a height that is more similar to that of "Lucy", the iconic skeleton of a female *A. afarensis*.

Looking back, 2016 has been a banner year for trace fossils in human evolution (e.g., Bennett et al., 2016; Liutkus-Pierce et al., 2016; Roach et al., 2016), and these new sets of footprints from Laetoli are a fitting capstone to the year. To judge by the profound scientific impact of the first set of Laetoli footprints, we can expect the new ones to figure prominently in future narratives of the origins of humans. They will likely stimulate new research and debate for years to come.
